# ESRRA-C11orf20 Is a Recurrent Gene Fusion in Serous Ovarian Carcinoma

**DOI:** 10.1371/journal.pbio.1001156

**Published:** 2011-09-20

**Authors:** Julia Salzman, Robert J. Marinelli, Peter L. Wang, Ann E. Green, Julie S. Nielsen, Brad H. Nelson, Charles W. Drescher, Patrick O. Brown

**Affiliations:** 1 Department of Biochemistry, Stanford University School of Medicine, Stanford, California, United States of America; 2 Department of Statistics, Stanford University, Stanford, California, United States of America; 3 Howard Hughes Medical Institute, Stanford University School of Medicine, Stanford, California, United States of America; 4 Fred Hutchinson Cancer Research Center, Seattle, Washington, United States of America; 5 Trev and Joyce Deeley Research Centre, BC Cancer Agency, Victoria, British Columbia, Canada; Medical Research Council, United Kingdom

## Abstract

Every year, ovarian cancer kills approximately 14,000 women in the United States and more than 140,000 women worldwide. Most of these deaths are caused by tumors of the serous histological type, which is rarely diagnosed before it has disseminated. By deep paired-end sequencing of mRNA from serous ovarian cancers, followed by deep sequencing of the corresponding genomic region, we identified a recurrent fusion transcript. The fusion transcript joins the 5′ exons of ESRRA, encoding a ligand-independent member of the nuclear-hormone receptor superfamily, to the 3′ exons of C11orf20, a conserved but uncharacterized gene located immediately upstream of ESRRA in the reference genome. To estimate the prevalence of the fusion, we tested 67 cases of serous ovarian cancer by RT-PCR and sequencing and confirmed its presence in 10 of these. Targeted resequencing of the corresponding genomic region from two fusion-positive tumor samples identified a nearly clonal chromosomal rearrangement positioning ESRRA upstream of C11orf20 in one tumor, and evidence of local copy number variation in the ESRRA locus in the second tumor. We hypothesize that the recurrent novel fusion transcript may play a role in pathogenesis of a substantial fraction of serous ovarian cancers and could provide a molecular marker for detection of the cancer. Gene fusions involving adjacent or nearby genes can readily escape detection but may play important roles in the development and progression of cancer.

## Abstract 2

Many ovarian cancers have a chromosomal rearrangement that fuses two neighboring genes, ESRRA and c11orf20. Similar rearrangements may be common, important features of cancer genomes that have largely escaped detection.

## Author Summary

Serous ovarian cancer, the most common form of ovarian cancer, is especially lethal because it is usually only detected at a late stage in its progression, after the cancer has spread to other tissues. We searched for molecular markers of this cancer that might provide a better way to detect tumors at a curable stage and that might provide targets for new treatments. Chromosomal rearrangements that fuse two genes to produce a recombinant gene that enhances growth or spread of the cancer are particularly specific biomarkers and have been found in many cancers. By “deep” sequencing of the RNA molecules that carry genetic information in serous ovarian cancers, we discovered a rearrangement that fuses the same two neighboring genes in at least 15% of these tumors. The two fused genes are ESRRA, which encodes a key regulator of gene expression, and an essentially uncharacterized gene, C11orf20, that is normally adjacent to the ESRRA gene. Chromosomal rearrangements that recombine parts of two nearby genes or even parts of a single gene may be a common, important feature of the cancer genome that eludes detection by most approaches to characterizing cancer genomes.

## Introduction

Ovarian cancer is estimated to kill more than 140,000 women every year [1]. Like most cancers, ovarian cancer has a dismal prognosis once the disease has spread beyond the site of origin [2]. The histological subtypes of ovarian cancer differ substantially in their molecular features and natural history and can be considered distinct diseases. Ovarian carcinomas of the serous histological type are responsible for the majority of deaths from ovarian cancer; they typically progress to an advanced stage while the tumor is still much too small to be detected by any presently available screening method [3]. Discovery of truly tumor-specific molecular markers may be essential for effective early detection of these tumors.

Recurrent gene fusions are among the most tumor-specific molecular markers known. Investigations of oncogenic gene fusions, including BCR-ABL in chronic myelogenous leukemia, have provided critical insights into pathogenesis and led to important therapeutic advances [4].

With a few notable exceptions, however, recurrent gene fusions have rarely been identified in commonly occurring carcinomas, which often have multiple, complex chromosomal rearrangements that are difficult to analyze by traditional cytogenetic approaches. A recurrent gene fusion, TMPRSS2-ERG, with an estimated prevalence of ∼50% in prostate cancer was discovered by a targeted search for cancer-associated genes with anomalous expression patterns, in a large database of DNA microarray data [5]. An ex vivo functional screen of cDNA from a non-small cell lung carcinoma (NSCLC) led to identification of EML4-ALK as a recurrent gene fusion in ∼5% of NSCLCs [6],[7].

Ultra High Throughput Sequencing (UHTS) is a powerful method for discovery of novel RNA sequences, including cancer-specific gene fusions. Tumor-specific genomic rearrangements and fusion transcripts have been discovered in individual tumors by UHTS (see for example [8]–[10]), including in high-grade serous ovarian cancer [11], but none of those reported to date have been recurrent. For example, a UHTS survey of genomic aberrations in 24 breast cancers found more than 2,000 rearrangements; 29 of these were predicted to generate in-frame gene fusions, but none occurred in more than one individual [12]. Similarly, a UHTS analysis of RNA from 10 melanomas identified 11 gene fusions, none of which were recurrent either in the original set or 90 additional cases [10].

We combined deep, paired-end sequencing of tumor RNA with a statistical bioinformatic approach to search for gene fusions in a pool of mRNA isolated from 12 primary serous ovarian cancers. Our analysis identified a novel recurrent gene fusion, ESRRA-C11orf20, resulting from a chromosomal rearrangement. The methods we used have important differences from previous algorithms for identifying gene fusions and novel splice variants [8]–[10], mainly in the use of statistical models for fusion discovery, and may be useful for discovering gene fusions in other cancers. (Note: since the algorithm used to identify the ESRRA-C11orf20 fusion was built, other algorithms for detecting fusions with RNA-Seq have been published [11],[13] with methods related to but algorithmically distinct from ours.)

## Results

### UHTS Analysis of RNA from Serous Ovarian Cancer Identifies a Candidate Gene Fusion

To search for recurrent or highly expressed fusion transcripts, we first prepared a cDNA library with an average insert size of 350 bp from a pool of 12 late-stage serous ovarian tumors. Using Illumina GA II instruments, we determined 30 million pairs of 76-nucleotide sequences and 80 million pairs of 38-nucleotide sequences from the ends of these cDNA segments, a total of 111 million purity filtered (PF) reads.

Our RNA sequence analysis pipeline is diagrammed in Figure S1. We began by identifying paired reads that mapped uniquely to any two distinct genes (call them genes A and B). However, most such paired reads are spurious, due to artifactual ligation during library preparation, sequencing errors, or paralogous sequences. We constructed a database of the sequences predicted for every possible exon-exon junction that might result from a fusion between such pairs of genes A and B in the RefSeq database. We then searched our sequence data for individual reads that failed to align to the RefSeq transcript database, but did align uniquely to a sequence in our database of hypothetical gene fusion exon-exon junctions (“junction reads”). To be considered further, we also required that any such sequence have at least 10 bp aligning to each side of the hypothetical junction and that its cognate paired-end read align to one of the corresponding fusion partners in an orientation consistent with the predicted A-B fusion (diagrammed in Figure S2). Rather than introduce more stringent filters to exclude potential artifacts, at the expense of discarding potentially important results, we used statistical models to estimate the distribution of falsely identified fusions and assess a false discovery rate (see Text S1).

**Figure 1:**
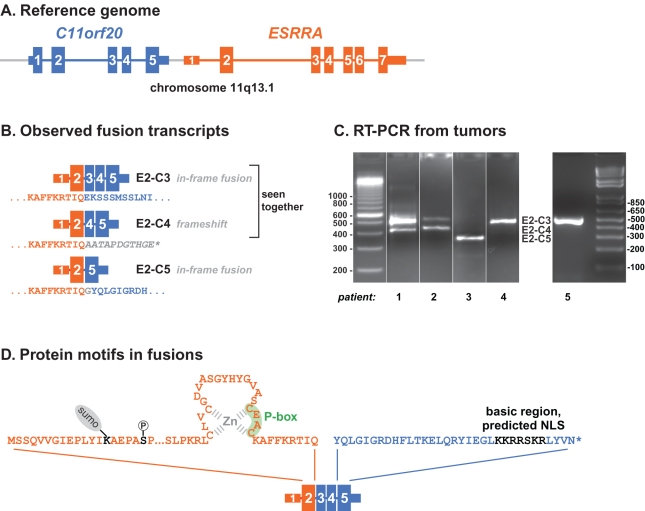
Fusion transcripts identified in serous ovarian cancers. (A) C11orf20 is an ORF transcribed from a region whose 5′ end is less than 1 kb upstream of ESRRA's transcriptional start in the wild-type genomic organization of 11q13.1. (B) Three isoforms of a fusion transcript, ESRRA-C11orf20, inconsistent with a wild-type genomic organization and canonical transcription have been detected by our sequence analysis of RNA from serous ovarian cancer cases. Each fusion isoform joins ESRRA exon 2 to a distinct exon of C11orf20. E2-C3 and E2-C5 are in frame events; E2-C4 is out of frame and has been detected in combination with E2-C3. (C) Representative RT-PCR reactions demonstrating the presence of the fusion in 5 individual cases. Patient 1–4 were from FHCRC and Patient 5 was from BCCA. Fusions were confirmed by Sanger sequencing; the specific fusion variants seen are detailed in Table S1. (D) All fusions are predicted to contain the N-terminal 108 amino acids of ESRRA, including the DNA-binding zinc finger and P-Box, and conserved phosphorylation and sumoylation sites (Ser 19, Lys 14, respectively); in-frame fusions all contain the C-terminal portion of C11orf20 with a basic putative nuclear-localization signal.

A transcript composed of exons from the ESRRA and C11orf20 genes was one of two putative fusion transcripts supported by more than three junction reads. (The other appeared to be a read-through transcript and has subsequently been annotated as RefSeq gene LOC100630923. The full list of candidates is given in Table S2.) Two distinct splice variants of a hypothetical fusion between ESRRA and C11orf20, joining exon 2 of ESRRA to either exon 3 or exon 4 of C11orf20 (E2-C3 and E2-C4, Figure 1B), were represented, E2-C3 with a low estimated false-discovery rate. We confirmed both of these predicted fusion transcripts by using RT-PCR to amplify the diagnostic exon junction sequences from pool RNA, followed by Sanger sequencing (Figure S3).

ESRRA (Estrogen Receptor Related Alpha, also known as ERR1) encodes a well-studied orphan nuclear receptor related to the estrogen receptor, and implicated in regulation of energy metabolism and thermogenesis, its expression level has been positively correlated with breast cancer progression and angiogenesis ([14]–[18]; review in [19]). In ovarian cancer, ESRRA expression has also been associated with decreased survival [20], and kaempferol, which inhibits angiogenesis by ovarian cancer cell lines, acts at least partially by decreasing ESRRA expression [21]. Very recently, the ESRRA locus has been implicated in increased risk of ovarian cancer [22]. By contrast, C11orf20 is a mostly uncharacterized gene, though conserved in the mammalian lineage.

Using single read count data [23], we estimated the expression level of ESRRA to be roughly 2500^th^ in abundance in our serous ovarian cancer pool, similar to the abundance, for example, of ESR1 (ranked ∼2700^th^) and TP53 (ranked ∼1700^th^). Based on a statistical model for mRNA isoforms in paired-end data [24], we estimated the relative abundance of the canonical ESRRA mRNA, the fusion transcript E2-C3, the fusion transcript E2-C4, and the canonical C11orf20 mRNA as 40∶10∶1∶0, respectively. The abundance of the fusion transcripts thus appeared to be comparable to or greater than that of the ESRRA transcript itself, in one or more tumors harboring the fusion. We found no evidence for expression of either the reciprocal fusion product or the predicted full-length C11orf20 transcript.

### Recurrence and Alternatively Spliced Variants of ESRRA-C11orf20

We evaluated the prevalence of the ESRRA-C11orf20 fusion in a set of 68 patients with serous ovarian cancer, by RT-PCR followed by Sanger sequencing. Nine of the 42 cases screened at the FHCRC and 1 of the 25 cases screened at the BCCA were fusion-positive (exemplary positive RT-PCRs in Figure 1C). This gives an estimated prevalence of ESRRA-C11orf20 fusion transcripts in serous ovarian cancer as 10 in 67, or 15% (exact binomial 95% confidence interval: 7% to 26%). It should be noted that, in order for a patient sample to be called fusion-positive, we required that the majority of technical PCR replicates be positive; some cases showed fusion products but less reproducibly and so our counts may be subject to false negatives; thus this prevalence may be an under-estimate.

Nearly all positive cases expressed one or both of the two ESRRA-C11orf20 fusion isoforms previously observed in our tumor pool (E2-C3, E2-C4). One patient expressed exclusively a third isoform (E2-C5) in which ESRRA exon 2 was spliced to exon 5 of C11orf20 (Patient 3, Figure 1C).

The ESRRA protein consists of an N-terminal regulatory domain (NTD), a DNA binding domain (DBD) comprising two zinc-fingers, and a putative ligand-binding domain (LBD) [19]. The fusion transcripts all encode the NTD and the first zinc-finger of the DBD, but lack both the second zinc-finger and the LBD. Two of the three fusion transcripts preserve reading frame across the junction (E2-C3 and E2-C5); both share sequences encoding the 35 C-terminal amino acids of the predicted C11orf20 protein, including a basic potential nuclear-localization signal (Figure 1D). The E2-C4 junction introduces a frameshift, resulting in a nonsense codon shortly after the junction (Figure 1B). All fusion-positive tumors we have identified expressed at least one of the in-frame isoforms.

### Genomic Rearrangement in the C11orf20-ESRRA Locus

In principle, the ESRRA-C11orf20 fusion transcripts could have resulted from: (1) an acquired or germline rearrangement of the C11orf20-ESRRA region of Chromosome 11 deviating from that in reported human reference genomes or reported variants (to our knowledge, no germline structural variant rearranging ESRRA and C11orf20's relative positions has been reported, including in the 1000 genomes project.), or (2) *trans*-splicing of ESRRA and C11orf20 transcripts from an unrearranged locus. To discriminate these possibilities, we used a hybridization-selection and UHTS strategy to deeply sequence the C11orf20-ESRRA genomic region in two tumors that were positive for the fusion transcripts E2-C3 and E2-C4. A matched normal PBMC sample was available for one tumor. We note in passing that all analyses presented here used original genomic DNA for library generation; initial attempts using phi29-amplified DNA gave apparently unreliable results.

Paired-end sequencing libraries were prepared from the three samples (a tumor/normal matched pair and one tumor lacking a matched normal). A 166 kb bacterial artificial chromosome (BAC) was used to enrich for reads in the ESRRA locus. The resulting enriched libraries were each sequenced in one lane of an Illumina GA IIx flowcell at an average sequencing depth of 8 million mapped 80 bp PE reads. The sequenced libraries all showed significant inhomogeneity in sequencing depth across the targeted interval (see Text S1); however, the inhomogeneous coverage was consistent between samples, allowing us to model copy number variation in the sequenced tumors by comparison to the normal PBMC sample.

**Figure 2:**
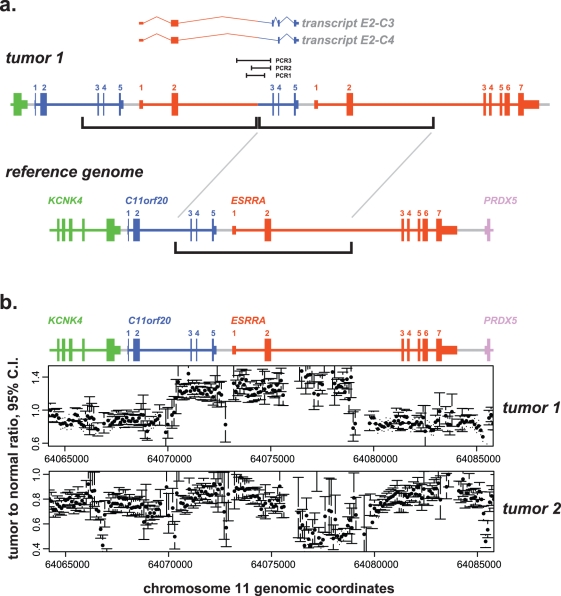
Genomic rearrangements in patient samples. Data for genomic structures of tumors analyzed with respect to the reference human genome, build hg19. C11orf20 and ESRRA regions are color-coded and exons numbered. KCNK4 is the gene immediately upstream of C11orf20 and PRDX5 is the gene immediately downstream of ESRRA. (A) Model for Tumor 1 DNA rearrangement. Brackets indicate a genomic interval (coordinates ch11∶64,070,517-64,079,032) from upstream of C11orf20 exon 3 to downstream of ESRRA exon 2, tandemly duplicated in the tumor (model shown above the reference). The transcripts (E2-C3 and E2-C4) expressed by the tumor are pictured above the rearrangement model, as are the extents of three PCR products spanning the breakpoint (PCR1, PCR2, PCR3). (B) Copy number plots for two tumors. The point estimate for copy number (as a dot) and 95% confidence interval (as error bars) are shown for each sequence interval (bin) with reliable counts. These were computed by grouping single aligned reads into 100 bp bins after removing potential PCR duplicates and comparing to the corresponding bin from the normal PBMC sample. For Tumor 1, copy number estimate interior to breakpoints is approximately 1.5× the copy number exterior to breakpoints, consistent with the model in (A), where one of two diploid chromosomes has a tandem duplication. For Tumor 2, inspection of the 95% error bars indicates that Tumor 2 exhibits statistically significant copy number variation within ESRRA intron 2, as well as at additional points.

Our genomic analysis strategy is summarized as follows and detailed in Text S1. Paired ends uniquely mapping to a 20 kb radius of ESRRA, whose joint chromosomal coordinates and orientations inconsistent with the reference genome were flagged. In Tumor 1, a statistically significant “pile” of PE reads predicted that ESRRA intron 2 had been rearranged upstream of C11orf20 exon 3. This hypothesis was tested using PCR to amplify the predicted rearrangement (PCR1, Figure 2A) and a computational method described below, both of which confirmed the expected breakpoint (sequence in Text S1).

Because the breakpoint in Tumor 1 is flanked by a SINE repeat both upstream in ESRRA and downstream in C11orf20, we performed additional PCRs using primers external to those in the first PCR, in parallel, on Tumor 1 DNA and negative control normal DNA, to rule out an in vitro PCR artifact. Each of these (PCR2, PCR3) produced a tumor-specific band of expected size, and the sequenced products showed the identical breakpoint.

In parallel with PCR confirmation, an unbiased computational approach using the de novo assembly program Velvet [25],[26] was used as a discovery tool (“orphan-end assembly”). Briefly, for each 200 bp window in the reference genome, all PE reads where one side aligned the reference in this window and the other side failed an alignment to the reference were flagged. The reads failing alignment were assembled using Velvet, and screened to determine if they supported a rearrangement placing ESRRA upstream of C11orf20. The breakpoint sequence discovered with PCR was also found using this computational method, and no other breakpoint providing a parsimonious explanation for an ESRRA-C11orf20 fusion was discovered in Tumor 1 or the other tumor (see Text S1). Furthermore, while Illumina library reads from Tumor 1 tiled the breakpoint, no Illumina sequence reads from any other library aligned to it.

Finally, copy number analysis of Tumor 1 (Figure 2B) shows a relative copy number increase precisely in the region between the reference coordinates defining the breakpoint (and nowhere else in the targeted region, analysis not shown). The simplest model to account for the junctional sequence and copy number data for Tumor 1 is that a tandem duplication of an interval between C11orf20 and ESRRA is present in one of two diploid copies of chromosome 1, as depicted in Figure 2A. Thus, sequence analysis provides strong evidence that the ESRRA-C11orf20 fusions in Tumor 1 are transcriptional products of a genomic rearrangement that positions ESRRA upstream of C11orf20 (rather than *trans*-splicing).

Tumor 2 shows significant copy number variation in the C11orf20 and ESRRA locus (Figure 2B), as well as a large degree of copy number variation throughout the region enriched by the BAC (analysis not shown). Although this is evidence for a genomic rearrangement in Tumor 2 in the critical region, we have not been able to pinpoint a breakpoint sequence with UHTS analysis for anomalously mapping read-pairs and orphan-end assembly, nor by long-range genomic PCR. Several types of rearrangements might not be detected by our short-read detection approach: for example, a complex rearrangement including ectopic sequence that does not hybridize to the BAC or a rearrangement within a region of ESRRA and C11orf20 that cannot be uniquely assigned to either gene. A substantial fraction of this region falls in a “blind spot” of this method: 10% of 80-mers in ESRRA (1,008 of 10,078) and 7% in C11orf20 (378 of 4,962) have more than 10 matches to the human genome (hg19 build).

## Discussion

We used UHTS analysis of RNA from a pool of tumor samples in a deliberate search for a recurrent gene fusion in serous ovarian cancer, a deadly cancer for which there is currently no early-detection screen and in which no recurrent gene fusions had been identified. Systematic analysis of the sequence data revealed novel fusion transcripts combining 5′ exons from ESRRA, a gene encoding a transcription factor of the nuclear hormone receptor family, and 3′ exons from C11orf20, an uncharacterized but phylogenetically conserved gene immediately upstream of ESRRA on Chromosome 11. In an RT-PCR/Sanger sequencing survey of serous ovarian cancers at two different institutions, we confirmed ESRRA-C11orf20 fusion transcripts in 10 of the 67 tumors, or 15% (95% confidence interval: 7% to 26%), suggesting that this fusion is present in a significant fraction of serous ovarian cancers.

To test the hypothesis that the ESRRA-C11orf20 fusion was the result of a genomic rearrangement, we combined hybridization selection of the C11orf20-ESRRA genomic region of Chromosome 11 with UHTS to analyze the structure of this interval in two tumors. The results provide strong evidence that a fusion transcript arose from a genomic rearrangement of the C11orf20-ESRRA region of Chromosome 11 in one tumor and copy-number variation evidence of rearrangement in the second tumor.

The ESSRA-C11orf20 fusion is, to our knowledge, the first recurrent gene fusion to be identified in serous ovarian cancer. This fusion gene and its components are now high-priority targets for further investigation of their potential roles in pathogenesis and as potential diagnostic or therapeutic targets. Our findings cast a spotlight on ESRRA as a candidate oncogene in serous ovarian cancer. ESRRA has been most studied in the context of breast cancer: it is a negative prognostic marker in ER(–) tumors [14],[15], and it induces VEGF mRNA expression and contributes to the malignant phenotype of a breast cancer cell line [16],[17]. It has been less studied in ovarian cancer, but has recently been associated with increased risk of ovarian cancer [22] and decreased patient survival [20].

Two of the three fusion isoforms we observed, E2-C3 and E2-C5, are in-frame and predicted to encode fusion proteins that contain the N-terminal portion of the ESRRA protein and the C-terminal portion of the predicted C11orf20 protein. Although one of the two zinc-finger domains and the putative ligand-binding domain of ESRRA are absent from the predicted fusion protein, important functional features of ESRRA are retained, including the first zinc-finger domain, critical for the DNA sequence specificity of ESRRA, as well as a phosphorylation site (Ser 19) and a phosphorylation-dependent sumoylation site (Lys 14) that have been shown to regulate transcriptional activation by ESRRA [19]. C11orf20 is a largely uncharacterized gene, with expression reportedly restricted to testis in mouse (RIKEN cDNA 1700019N12; [27]) and human (http://biogps.gnf.org). The predicted protein product of C11orf20 is conserved in mammals but uncharacterized; it lacks any known functional domains and has no apparent homology to any protein of known function.

Although any functional role for the ESSRA-C11orf20 fusion remains to be established, fusions to other nuclear hormone receptor transcription factors have been found in other cancers, including PAX8–PPARG in follicular thyroid tumors [28], EWSR1-NR4A3 in extraskeletal myxoid chondrosarcomas [29], and PML-RARA in acute promyelocytic leukemia [30]. In those fusions the nuclear receptor portion comprises the C-terminal component of the fusion protein and contains the entire DNA-binding and ligand-binding domains, whereas in the fusions reported here, the ESRRA component is N-terminal and contains only the first half of the DNA-binding domain (P-box zinc finger). Single zinc fingers, however, can mediate DNA-binding, for example in GATA-1 and SUPERMAN; in these known examples, adjacent basic regions are also required [31],[32]. It is therefore noteworthy that the in-frame fusions we identified join the ESRRA P-box zing finger to a basic sequence in the C11orf20 C-terminus (Figure 1).

We have presented evidence that a tumor-specific ESRRA-C11orf20 fusion transcript is present in a substantial fraction of serous ovarian cancers and that in one of two profiled tumors, Tumor 1, a genomic rearrangement that can account for the fusion transcript is present in a majority of tumor cells. Copy number variation at the ESRRA locus also suggests a structural rearrangement in Tumor 2. Although it remains possible that the ESRRA-C11orf20 fusion is an incidental consequence of another, functionally important, genetic event or that it is merely a “passenger,” the apparent frequency with which this rearrangement occurs in serous ovarian cancer and the lack of evidence that it accompanies large-scale structural variation (such as gene amplification) are more suggestive of a direct role.

Several characteristics of the ESRRA-C11orf20 rearrangement reinforce themes emerging from high-resolution studies of both normal human genetic variation [33],[34] and cancer-specific genomic alterations. Indeed, although none were found to be recurrent, 4 of the 11 gene fusions identified in a previous UHTS survey of RNA from a series of melanomas were strikingly similar to the ESRRA-C11orf20 fusion; adjacent genes in the same transcriptional orientation were rearranged to yield a fusion transcript in which the order of the two participating genes was reversed [10]. In a second study, using UHTS to profile genomic rearrangements in 24 breast cancers, the overwhelming majority of rearrangements identified were intrachromosomal; more than 90% of these involved breakpoints separated by 2 Mb or less [12]. These rearrangements, like the ESRRA-C11orf20 rearrangement described here, are consistent with a model in which double-strand breaks are preferentially repaired by joining sequences in physical proximity [35]–[37]. Most such fine-scale genomic rearrangements, including the ESRRA-C11orf20 rearrangement, cannot be detected by traditional cytogenetic methods, nor, unless they lead to extensive copy-number alterations, by array CGH. “Exome sequencing” will generally fail to detect any chromosomal rearrangement, except for the rare cases in which a breakpoint falls within an exon. A very recent large integrated genomics survey indeed found that high-grade serous ovarian carcinoma is characterized by a higher degree of somatic copy-number alterations and lower degree of somatic point mutations than the previously surveyed cancer glioblastoma [38]; however, the methods employed were unlikely to (and did not) identify the rearrangement presented here. We were able to detect the ESRRA-C11orf20 fusion, based on UHTS analysis of either RNA or genomic DNA, only by conducting a deliberate focused search for evidence of structural rearrangements. We suggest that chromosomal rearrangements involving nearby or adjacent genes may comprise a substantial fraction of oncogenic mutations that have heretofore escaped detection.

## Materials and Methods

### Specimen Collection

Ovarian cancer samples and matched controls were collected following procedures approved by the IRB at each institution: from the Pacific Ovarian Cancer Research Consortium (POCRC) and Fred Hutchinson Cancer Research Center (FHCRC), and from the British Columbia Cancer Agency (BCCA) Tumour Tissue Repository, Victoria, BC, a member of the Canadian Tumour Repository Network. Samples were (1) collected at initial debulking surgery using standardized protocols and (2) reviewed by a gynecological research pathologist to confirm the histological characteristics of the tissue; all tumor samples used in this article contained at least 70% malignant epithelium. Clinical data for RT-PCR screened samples are shown in Table S1.

### RNA-SEQ Library Preparation

RNA was pooled from 12 high grade serous stage III/IV carcinoma of the ovary samples together with doping control RNA (see Text S1). 10 micrograms total RNA was diluted with water to 50 microliters, heated to 70 °C for 5 min, and purified with DYNAL DynaBeads Oligo (dT)_25_ (Invitrogen, Carlsbad, CA, USA) per manufacturer protocol. RNA was fragmented to an average size of 350 bp by alkaline hydrolysis: RNA was added to preheated fragmentation buffer (50 mM sodium carbonate/bicarbonate, 1 mM EDTA, pH 9.2) and incubated at 95 °C for 6 min, then neutralized with 1/10 volume of 3 M sodium acetate pH 5.2, and precipitated with 3 volumes ice-cold EtOH. The pellet was washed with 75% EtOH, dried, and resuspended in water.

First and second strand cDNA synthesis, end repair, 3′-dA tail addition, and paired-end adaptor ligation were performed using standard protocols and reagents from the PAIRED-END Sample Prep Kit (part # 1001809, Illumina, San Diego, CA, USA). cDNA products were resolved by electrophoresis in 2% low-melting agarose gels, one sample per gel. The gels were stained with SYBR Gold (Invitrogen) and visualized on a blue light table (Dark Reader, Clare Chemical Research, Dolores, CO). The desired band was excised with sterile scalpels and purified with a QIAquick Gel Extraction kit with the modifications described in [39] to minimize GC-bias. Each sample was amplified with Phusion DNA Polymerase and Illumina primers PE 1.0 and PE 2.0 for 15 cycles, then purified with a QIAquick PCR purification kit per Illumina library preparation protocol.

The concentration of each sample was determined using an Agilent Bioanalyzer. Samples were then diluted to a concentration of 10 pM as specified by Illumina protocols. The sample derived from pooled tumor RNA was subjected to 76-base, paired-end sequencing in two lanes of an Illumina Genome Analyzer II and, in a separate run, 7 lanes of 38-base paired-end sequencing. Sequencing runs all used the Illumina Sequencing Kit v3-36 reagents. Sequencing data from this study are available on the SRA through dbGaP.

### Selection of Fusion Candidates from Paired End Reads

As seen in Figure S1, reads from two 76-base, paired-end lanes and seven 38-base, paired-end lanes were passed through the Illumina PF filter and aligned using Bowtie [40] to the hg19 RefSeq transcriptome as paired-end reads. Those paired ends that successfully aligned were put aside as they do not represent novel fusion events. The paired-end sequences that failed this alignment were then subjected to alignments of each end separately with a more stringent requirement for unique alignment within the RefSeq transcriptome. Paired reads, of which side 1 mapped uniquely to a RefSeq annotated gene (gene A) and side 2 mapped uniquely to a different RefSeq annotated gene (gene B), were taken as indirect evidence of a fusion between gene A and gene B. A FASTA file of all exon-exon junctions between gene A and gene B was generated; reads that failed to align to the reference transcriptome were aligned to this FASTA file of exon-exon junctions. 76-mer reads that aligned to a junction between genes A and B, including at least 10 bp overlap on each side of the junction, and whose mate mapped to either gene A or gene B, were subjected to further analysis.

### RT-PCR Validation and Screening

cDNA was prepared with SuperScript III First-Strand Synthesis kit, PCR amplifications were performed with Platinum Taq DNA Polymerase, and products were cloned with TOPO TA Cloning kits, all from Invitrogen (Carlsbad, CA, USA).

For initial RT-PCR validation in the RNA pool, we used primers G1P1-FWD  =  5′-GGCATTGAGCCTCTCTACATCA-3′ (ESRRA exon 2) and G2P1-REV  =  5′-TCGATGTATCGCTGCAGCTCCTTA-3′ (C11orf20 exon 5). PCR was run for 40 cycles; each cycle was 94°C 15 s, 55°C 30 s, 70°C 60 s.

For screening of fusion transcript prevalence, we used a nested RT-PCR for increased specificity. For each sample, we performed up to 6 technical replicates, and only considered positive if a majority of replicates gave a fusion product. The outer primers were G1P1-FWD  =  5′-GGCATTGAGCCTCTCTACATCA-3′ (ESRRA) and REV_pair3  =  5′-GGGTCAGGCTTGGGTCTG-3′ (C11orf20); the inner primers were G1P2-FWD  =  5′-AAAGGGTTCCTCGGAGACAGAGA-3′ (ESRRA) and F1-REV  =  5′-TAATTCACGTACAGCCTCTTGCTCCG-3′ (C11orf20). The outer PCR was run for 20 cycles, then diluted 1/200 into inner PCR mix, and run for 30 cycles; each cycle was 94°C 15 s, 55°C 30 s, 72°C 60 s.

### Hybrid-Selection and UHTS of Genomic DNA:

Tissue samples were obtained from two FHCRC patients whose tumor samples expressed the ESRRA-C11ORF20 fusion transcript (one tumor lacked a matched normal). The samples were processed using TRIZOL (Invitrogen) to extract RNA and genomic DNA.

The DNA samples were sheared to an intended size of 400 bp in Covaris sample tubes (part # 500111; Covaris, Inc., Woburn, MA, USA) in a Covaris S2 controlled by SonoLab software, using settings of 10% duty cycle, intensity 4, 200 cycles per burst, for two 30-s periods.

We generally followed the Illumina protocol for hybridization enrichment libraries, using Herculase II Fusion Enzyme (Agilent, Santa Clara, CA, USA) for PCR amplification. Samples were purified between steps using Agencourt AMPure SPRI XP beads (Beckman Coulter, Brea, CA, USA).

Hybrid-selection of the Illumina genomic libraries was based on [41]–[43]. A fully sequenced BAC, RP11-783K16 (GenBank # AP001453) encompassing the C11orf20-ESRRA region, was obtained from BACPAC Resource Center (Oakland, CA). BAC DNA was biotinylated using a nick-translation kit (Roche Applied Science, Indianapolis, IN). Illumina library (0.8 micrograms) was hybridized at 65 °C for >24 h to biotinylated BAC DNA (0.2 micrograms) in a solution containing: Cot-1 DNA (4 micrograms), sheared *E. coli* DNA (1 microgram), sheared vector DNA (0.5 micrograms), four adaptor-blocking oligos ([43]; 600 pmoles each), in 5× SSPE, 5 mM EDTA, 5× Denhardt's, 0.1% SDS (total volume 24 microliters). The genomic library DNA that hybridized to the BAC probe was captured on streptavidin-magnetic beads (Dynal MyOne, Invitrogen), which were then washed and eluted with 0.1 M NaOH. The eluate was precipitated and resuspended in 60 microliters of water. 20 microliters of the resulting solution of hybridization-selected genomic library DNA was amplified with Illumina PCR primers for 18 cycles (within the exponential amplification range), yielding ∼1 microgram of product. Each hybridization-selected genomic DNA library was sequenced on a separate lane of an Illumina GAIIx flow cell.

### Genomic Sequence Analysis

We identified read-pairs in the selected region where the distance between the paired sequences in the reference genome was greater than 1 kb—inconsistent with library insert sizes (which were <0.8 kb). The C11ORF20-ESRRA genomic region was divided into bins. The counts of anomalous read-pairs were compiled in a 2-dimensional histogram where each axis represented the genomic coordinate (bin) of one end of the read-pair, with read 1 aligning in the (+) orientation and read2 aligning in the (−) orientation. This was done for each sample, both tumors and normals. Pile-ups were nominated for further consideration at a given false discovery rate using a Poisson model for PE reads that takes into account position-specific bias. This model and subsequent analysis is detailed in Text S1.

The following computational approach was implemented to discover highly represented sequences inconsistent with the reference. A 20 kb radius around the transcriptional start of ESRRA was discretized into 200 bp bins. For each bin, reads where one read aligned to the plus strand and the other read failed to align to the human genome hg19 build were flagged, and the unaligned reads were consolidated and input to the de novo assembler Velvet. The same procedure was followed for reads where one read aligned to the minus strand. Velvet outputs contigs: putative sequences assembled from input reads. These contigs were subjected to further analysis by computationally fragmenting each contig to tiling 80-mers and aligning these 80-mers to the genome. In order to narrow our search to tumor-specific rearrangements, only contigs with portions that failed to align to the reference genome were scrutinized. Contigs which had sample-specific representation in the sequencing data (i.e., present in one tumor, and none of the remaining samples, or present in the normal sample of one individual and none of the remaining samples) were further scrutinized. The only such sequence with the potential to directly explain a genomic configuration capable of generating the fusion transcript was found in Tumor 1 and confirmed by PCR (see Text S1). Sequencing data from this study are available on the SRA through dbGaP.

## Supporting Information

Figure S1 Detailed analysis pipeline for detection of fusion transcripts in paired-end sequences from tumor RNA. The pipeline for analysis of sequences from tumor RNA is schematized with files in blue and Postgres tables in red. We start by aligning paired-end reads to RefSeq using Bowtie, retaining reads which failed to align (leftovers) in table read1 leftovers and read2 leftovers. The leftovers are re-aligned separately to RefSeq using Bowtie with m  =  1 (unique) and alignments retained as r1seq and r2seq Postgres tables. We identify mate pairs in these alignments where one gene (A) differs from the paired mate (B). We then created a database of all A-B, B-A, A-A, and B-B junctions. We created junctions using all the exons in each gene A and B from the mate pair A-B as well as the exons within A and the exons within B. All long (76 bp) purity filtered (PF) reads were then aligned to the junction database, and successful alignments were tracked by Postgres tables. We performed queries to select reads with a transcriptome alignment as one half of a mate pair, and a junction read-through on the other mate, resulting in a table of fusion candidates.
(EPS)

Figure S2 Null hypothesis (fusions explained by homology) versus alternative hypothesis (potentially real fusion). (A) Orientation of alignments of Paired End (PE) reads from potentially real fusions at exon-exon boundaries. (B) Orientation of alignments of PE reads from putative fusions arising from homology between gene 1 and gene 2 at exon-exon boundaries. An intra-gene read that matches to a fusion junction due to sequence homology (2nd step) can be interpreted as evidence for a fusion, but has a polarity inconsistent with the gene order in the fusion.
(EPS)

Figure S3 RT-PCR fusion products seen in the Ovarian Cancer 12 patient pool. Lanes 1 and 2 are beta-Actin controls, expected 353 bp product. Lane 3 is a negative beta-Actin control. Lanes 4 through 7 are fusion products. Lanes 4 and 6 RT used gene specific primers G2P1-REV and G2P2-REV. Lanes 5 and 7 RT used oligo(dT) primer. Lanes 4 and 5 PCR primers are G1P1-FWD and G2P1-REV “pair-1.” Lanes 6 and 7 PCR primers are G1P2-FWD and G2P2-REV “pair-2.” Lane 8 is a negative H_2_O control. Lanes 9 and 10 are ladder: 100 bp, 250 bp, 400 bp, 800 bp, and 1,500 bp. Primer sequences and predicted product sizes are given in Text S1.
(TIFF)

Table S1 Clinical samples. Clinical details (age, stage, grade, histology, and chemotherapy) are shown for (1) the 42 patients from FHCRC and (2) the 25 patients from BCCA. Fusion-positive cases are indicated by shading and the isoforms identified are given in the column “Observed Fusion Isoforms.”
(XLS)

Table S2 Potential fusion candidates. The fusion candidates derived from our RNA pipeline are listed, sorted by the first column “Count,” which sums all the junctional reads involving a given pair of genes. For each gene pair, the precise exons fused are listed; when more than one distinct exon-exon junction was identified, all are listed. In some cases of short exons, reads matched to consecutive exons in a gene: for example “COL1A1.exon10,11:COL1A2.exon1” indicates a match involving both exon 10 and 11 of COL1A1 as the 5′ side of the fusion. The ESRRA-C11orf20 fusion studied in detail in this report has rank 2 in the list, with five junctional counts; four correspond to the E2-C3 isoform and one to the E2-C4 isoform.
(XLS)

Text S1 Supporting text.
(DOC)

## References

[1] Garcia M, Jemal A, Ward E. M, Center M. M, Hao Y, Society A. C (2007). Global cancer facts & figures 2007.. Atlanta: American Cancer Society.

[2] Kosary C, Ries L. A. G, Young J. L, Keel G. E, Eisner M. P, Lin Y. D (2007). Cancer of the ovary.. SEER survival monograph: cancer survival among adults: US SEER Program, 1988-2001, patient and tumor characteristics.

[3] Brown P. O, Palmer C (2009). The preclinical natural history of serous ovarian cancer: defining the target for early detection.. PLoS Med.

[4] Druker B. J, Talpaz M, Resta D. J, Peng B, Buchdunger E (2001). Efficacy and safety of a specific inhibitor of the BCR-ABL tyrosine kinase in chronic myeloid leukemia.. N Engl J Med.

[5] Tomlins S. A, Rhodes D. R, Perner S, Dhanasekaran S. M, Mehra R (2005). Recurrent fusion of TMPRSS2 and ETS transcription factor genes in prostate cancer.. Science.

[6] Rikova K, Guo A, Zeng Q, Possemato A, Yu J (2007). Global survey of phosphotyrosine signaling identifies oncogenic kinases in lung cancer.. Cell.

[7] Soda M, Choi Y. L, Enomoto M, Takada S, Yamashita Y (2007). Identification of the transforming EML4-ALK fusion gene in non-small-cell lung cancer.. Nature.

[8] Maher C. A, Kumar-Sinha C, Cao X, Kalyana-Sundaram S, Han B (2009). Transcriptome sequencing to detect gene fusions in cancer.. Nature.

[9] Maher C. A, Palanisamy N, Brenner J. C, Cao X, Kalyana-Sundaram S (2009). Chimeric transcript discovery by paired-end transcriptome sequencing.. Proc Natl Acad Sci U S A.

[10] Berger M. F, Levin J. Z, Vijayendran K, Sivachenko A, Adiconis X (2010). Integrative analysis of the melanoma transcriptome.. Genome Res.

[11] McPherson A, Hormozdiari F, Zayed A, Giuliany R, Ha G (2011). deFuse: an algorithm for gene fusion discovery in tumor RNA-seq data.. PLoS Comput Biol.

[12] Stephens P. J, McBride D. J, Lin M. L, Varela I, Pleasance E. D (2009). Complex landscapes of somatic rearrangement in human breast cancer genomes.. Nature.

[13] Sboner A, Habegger L, Pflueger D, Terry S, Chen D. Z (2010). FusionSeq: a modular framework for finding gene fusions by analyzing paired-end RNA-sequencing data.. Genome Biol.

[14] Ariazi E. A, Clark G. M, Mertz J. E (2002). Estrogen-related receptor alpha and estrogen-related receptor gamma associate with unfavorable and favorable biomarkers, respectively, in human breast cancer.. Cancer Res.

[15] Riggins R. B, Mazzotta M. M, Maniya O. Z, Clarke R (2010). Orphan nuclear receptors in breast cancer pathogenesis and therapeutic response.. Endocr Relat Cancer.

[16] Stein R. A, Chang C. Y, Kazmin D. A, Way J, Schroeder T (2008). Estrogen-related receptor alpha is critical for the growth of estrogen receptor-negative breast cancer.. Cancer Res.

[17] Stein R. A, Gaillard S, McDonnell D. P (2009). Estrogen-related receptor alpha induces the expression of vascular endothelial growth factor in breast cancer cells.. J Steroid Biochem Mol Biol.

[18] Villena J. A, Hock M. B, Chang W. Y, Barcas J. E, Giguere V (2007). Orphan nuclear receptor estrogen-related receptor alpha is essential for adaptive thermogenesis.. Proc Natl Acad Sci U S A.

[19] Tremblay A. M, Giguere V (2007). The NR3B subgroup: an ovERRview.. Nucl Recept Signal.

[20] Sun P, Sehouli J, Denkert C, Mustea A, Konsgen D (2005). Expression of estrogen receptor-related receptors, a subfamily of orphan nuclear receptors, as new tumor biomarkers in ovarian cancer cells.. J Mol Med.

[21] Luo H, Rankin G. O, Liu L, Daddysman M. K, Jiang B. H (2009). Kaempferol inhibits angiogenesis and VEGF expression through both HIF dependent and independent pathways in human ovarian cancer cells.. Nutr Cancer.

[22] Permuth-Wey J, Chen Y. A, Tsai Y. Y, Chen Z, Qu X (2011). Inherited variants in mitochondrial biogenesis genes may influence epithelial ovarian cancer risk.. Cancer Epidemiol Biomarkers Prev.

[23] Mortazavi A, Williams B. A, McCue K, Schaeffer L, Wold B (2008). Mapping and quantifying mammalian transcriptomes by RNA-Seq.. Nat Methods.

[24] Salzman J, Jiang H, Wong W. H (2011). Statistical modeling of RNA-Seq data.. Statistical Science.

[25] Zerbino D. R, Birney E (2008). Velvet: algorithms for de novo short read assembly using de Bruijn graphs.. Genome Res.

[26] Zerbino D. R, McEwen G. K, Margulies E. H, Birney E (2009). Pebble and rock band: heuristic resolution of repeats and scaffolding in the velvet short-read de novo assembler.. PLoS One.

[27] Kogo H, Kowa-Sugiyama H, Yamada K, Bolor H, Tsutsumi M (2010). Screening of genes involved in chromosome segregation during meiosis I: toward the identification of genes responsible for infertility in humans.. J Hum Genet.

[28] Kroll T. G, Sarraf P, Pecciarini L, Chen C. J, Mueller E (2000). PAX8-PPARgamma1 fusion oncogene in human thyroid carcinoma [corrected].. Science.

[29] Clark J, Benjamin H, Gill S, Sidhar S, Goodwin G (1996). Fusion of the EWS gene to CHN, a member of the steroid/thyroid receptor gene superfamily, in a human myxoid chondrosarcoma.. Oncogene.

[30] Zelent A, Guidez F, Melnick A, Waxman S, Licht J. D (2001). Translocations of the RARalpha gene in acute promyelocytic leukemia.. Oncogene.

[31] Dathan N, Zaccaro L, Esposito S, Isernia C, Omichinski J. G (2002). The Arabidopsis SUPERMAN protein is able to specifically bind DNA through its single Cys2-His2 zinc finger motif.. Nucleic Acids Res.

[32] Omichinski J. G, Clore G. M, Schaad O, Felsenfeld G, Trainor C (1993). NMR structure of a specific DNA complex of Zn-containing DNA binding domain of GATA-1.. Science.

[33] Itsara A, Wu H, Smith J. D, Nickerson D. A, Romieu I (2010). De novo rates and selection of large copy number variation.. Genome Res.

[34] Pang A. W, MacDonald J. R, Pinto D, Wei J, Rafiq M. A (2010). Towards a comprehensive structural variation map of an individual human genome.. Genome Biol.

[35] Ferguson D. O, Sekiguchi J. M, Chang S, Frank K. M, Gao Y (2000). The nonhomologous end-joining pathway of DNA repair is required for genomic stability and the suppression of translocations.. Proc Natl Acad Sci U S A.

[36] Pace J. K, Sen S. K, Batzer M. A, Feschotte C (2009). Repair-mediated duplication by capture of proximal chromosomal DNA has shaped vertebrate genome evolution.. PLoS Genet.

[37] Soutoglou E, Dorn J. F, Sengupta K, Jasin M, Nussenzweig A (2007). Positional stability of single double-strand breaks in mammalian cells.. Nat Cell Biol.

[38] The Cancer Genome Atlas Research Network (2011). Integrated genomic analyses of ovarian carcinoma.. Nature.

[39] Quail M. A, Kozarewa I, Smith F, Scally A, Stephens P. J (2008). A large genome center's improvements to the Illumina sequencing system.. Nat Methods.

[40] Langmead B, Trapnell C, Pop M, Salzberg S. L (2009). Ultrafast and memory-efficient alignment of short DNA sequences to the human genome.. Genome Biol.

[41] Bashiardes S, Veile R, Helms C, Mardis E. R, Bowcock A. M (2005). Direct genomic selection.. Nat Methods.

[42] Gnirke A, Melnikov A, Maguire J, Rogov P, LeProust E. M (2009). Solution hybrid selection with ultra-long oligonucleotides for massively parallel targeted sequencing.. Nat Biotechnol.

[43] Hodges E, Rooks M, Xuan Z, Bhattacharjee A, Benjamin Gordon D (2009). Hybrid selection of discrete genomic intervals on custom-designed microarrays for massively parallel sequencing.. Nat Protoc.

