# Supplementary figures and images for: ESRRA-C11orf20 Is a Recurrent Gene Fusion in Serous Ovarian Carcinoma
Source: PLoS Biol. 2011 Sep 20;9(9):e1001156. doi: 10.1371/journal.pbio.1001156 (PMC3176749; doi:10.1371/journal.pbio.1001156)

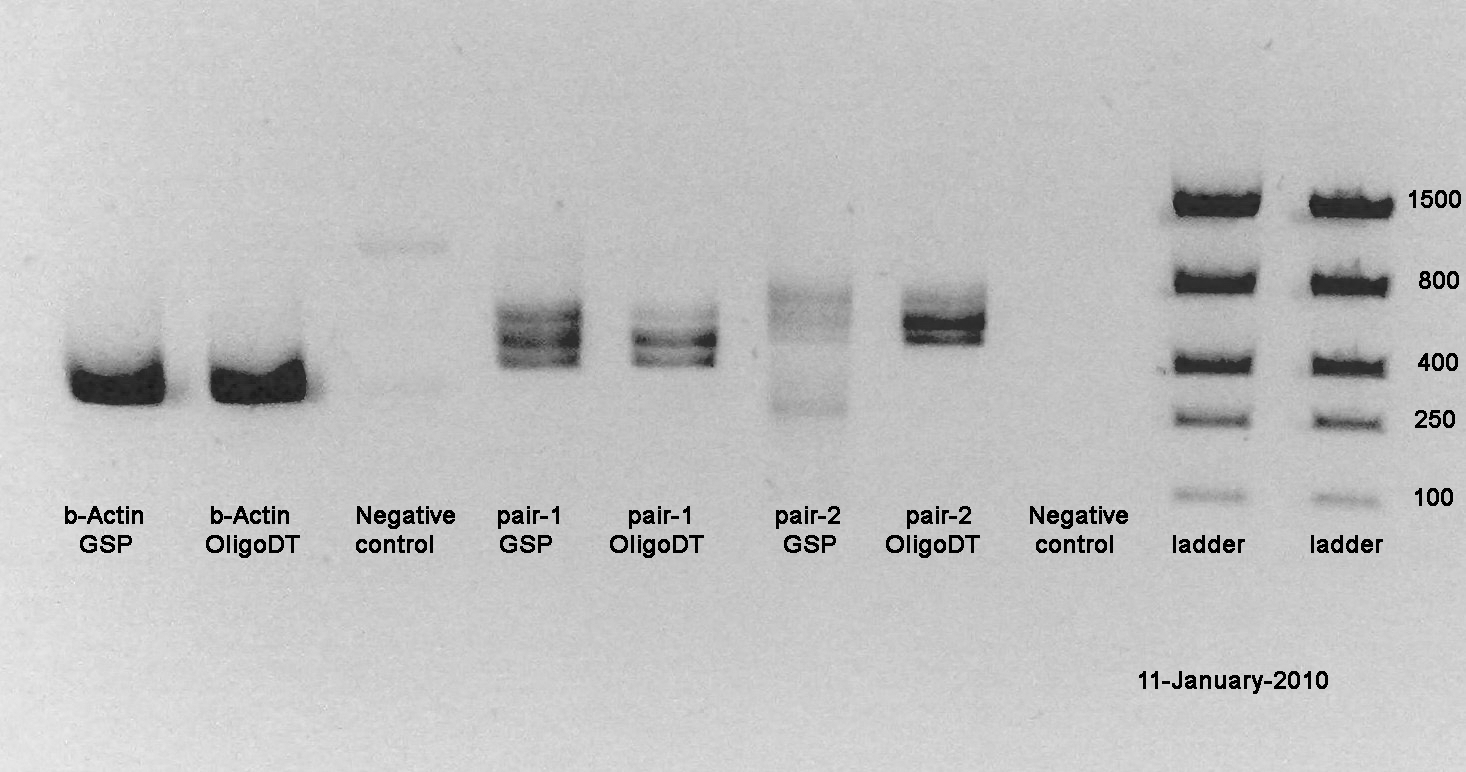

Supplement: Figure S3 — RT-PCR fusion products seen in the Ovarian Cancer 12 patient pool. Lanes 1 and 2 are beta-Actin controls, expected 353 bp product. Lane 3 is a negative beta-Actin control. Lanes 4 through 7 are fusion products. Lanes 4 and 6 RT used gene specific primers G2P1-REV and G2P2-REV. Lanes 5 and 7 RT used oligo(dT) primer. Lanes 4 and 5 PCR primers are G1P1-FWD and G2P1-REV “pair-1.” Lanes 6 and 7 PCR primers are G1P2-FWD and G2P2-REV “pair-2.” Lane 8 is a negative H2O control. Lanes 9 and 10 are ladder: 100 bp, 250 bp, 400 bp, 800 bp, and 1,500 bp. Primer sequences and predicted product sizes are given in Text S1. (TIFF) [file pbio.1001156.s003.tiff]
