# Supplementary material for: ESRRA-C11orf20 Is a Recurrent Gene Fusion in Serous Ovarian Carcinoma
Source: PLoS Biol. 2011 Sep 20;9(9):e1001156. doi: 10.1371/journal.pbio.1001156 (PMC3176749; doi:10.1371/journal.pbio.1001156)
Supplement: Text S1 — Supporting text. (DOC) [file pbio.1001156.s006.doc]

**Text S1**

***Contents***

**1. RNA-Seq**

a) Doping Controls for RNA-Seq Library Prep

b) RNA-Seq Fusion Detection Algorithm and Statistical Methods

c) Fusion Candidate List

d) ESRRA-C11orf20 Fusion Sequences

e) RT-PCR from the RNA pool: Primers and Product Sizes

**2. Genomic Analysis**

a) Overview of Sequencing Samples

b) Sequencing Enrichment and Coverage of the Region Of Interest (ROI)

c) Read Coverage Bias in Hybridization-Selected Libraries

d) Candidate rearranged regions from statistically anomalous read-pair distributions

e) Limited Assembly using Velvet

f) Statistical Copy Number Modeling

**3. DNA rearrangement:**

a) Tumor 1 breakpoint and flanking sequence from three sets PCR primers

*note:* all genomic coordinates are based on the Feb. 2009 (GRCh37/hg19) genome, accessed via the UCSC Genome Browser.

***1. RNA-Seq***

**a) Doping Controls for RNA-Seq Library Prep**

We prepared a pool of RNA from twelve patient samples as detailed in Table S1. We added control RNA sequences selected from NCBI GenBank DQ516718 through DQ516909, which are derived from from the *Methanocaldococcus jannaschii* genome and originally developed for use as RNA Spike-in Controls for calibration and QC of DNA microarray hybridizations. These “MJ” RNAs range in length from 125 bases to 8000 bases; Bowtie paired-end alignment (m=1) resulted in 88.7% transcriptome alignments, 3.6% MJ alignments and 7.7% rDNA alignments.

**b) RNA-Seq Fusion Detection Algorithm and Statistical Methods**

The RNA-Seq fusion detection algorithm is shown as flowcharts in Figure S1. In Figure S2, blue represents files and red represents Postgres Structured Query Language (SQL) tables.

**Algorithm Outline**

1) Align paired-end (PE) Reads to RefSeq, retaining leftovers which fail to align

2) Align leftovers separately to RefSeq with unique alignment

3) Identify gene A gene B PE reads and create database of all A-B, B-A, A-A and B-B junctions

4) Align all reads to junction library

5) Query database for reads with unique alignments to A-B or B-A junctions, and whose mate maps to A or B

*1) Align PE Reads to RefSeq, retaining leftovers that fail to align*

The paired-end purity-filtered reads were passed to Bowtie requesting a paired-end alignment, m=1 (unique alignments only) against the hg19 transcriptome from UCSC plus the MJ spike-in sequences and sequences for ribosomal DNA (rDNA), retaining the valid paired end alignments as Postgres table r12seq. Those reads failing to produce paired end alignments (leftovers) were written to a read1 leftovers file and a read2 leftovers file.

*2) Align leftovers separately to RefSeq with unique alignment*

Those read1 and read2 leftovers were then independently re-aligned to the transcriptome+MJ+rDNA as above, and the alignments retained as r1seq and r2seq again as Postgres tables.

*3) Identify gene A-gene B PE reads and create database of all A-B, B-A, A-A and B-B junctions*

A FASTA file of all possible junctions between pairs of exons of all gene pairs obtained from the alignments (step 2), named by the two gene+exon(s) comprising each junction, each of length 132 bases (66 bases of each gene). Our goal was to find pairs of 76-mer reads in which where one side of the pair had a valid transcriptome alignment and the other side aligned to one of the computed junctions; the lengths enforce that a matching read must have at least 10 base overlap to each gene in the junction. For each gene pair used to produce junctions, we also included all intra-gene junctions for each of the genes; as we required unique alignments to these junctions, we nominally discriminate against putative exon junctions that might have derived from either gene of the pair, though some such read-pairs pass this filter, presumably due to sequencing errors. We produced an alignable junction library from the FASTA records.

*4) Align all reads to junction library*

The original long (76 bp) purity-filtered reads were aligned to the above junction library, the successful alignments being tracked by Postgres tables.

*5) Query database for reads with unique alignments to A-B or B-A junctions, and whose mate maps to A or B*

We performed SQL queries to retrieve candidate fusions represented by a good alignment of one read to “gene A” and a good alignment of the second of the paired reads to a putative “gene A-gene B” exon junction.

**Statistical testing of junction alignment methods**

We assessed our methods for junction alignment against some possible null statistical models.

One null model we considered was that junctional hits were the result of homologous degenerate sequences at the boundaries of exons, in which case junction offsets would predominantly correspond to smallest allowed overlaps with the second (presumably spuriously identified) gene, and their frequency would decrease with the length of the putative overlap. We assessed whether a 10 bp overlap to each gene was sufficient to identify a lack of fit to a null model that the number of reads mapping at offset *i* should be proportional to *x^i^* with *x* < 1. The actual data for junction offsets, with PE read-lengths of both 38 and 76 bp, does not fit this null model (not monotonically decreasing from midpoint), as shown in the following plots:


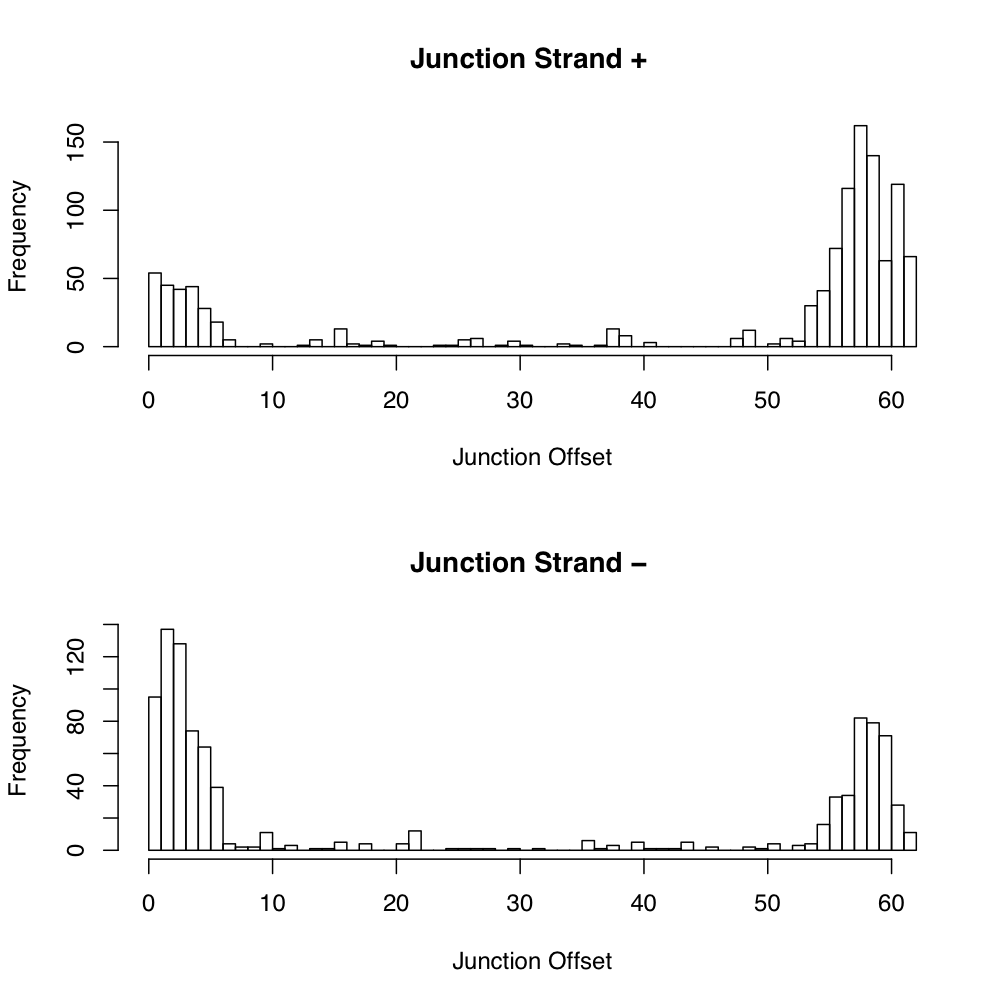


A second null model considered was that junction hits were all chimeras formed by inter-molecular ligation of two cDNAs (with no sequence dependence). This model predicts a uniform distribution of junction offsets at a much lower frequency than observed. We can estimate the frequency of chimera formation as ~5% [Quail MA *et al.*, *Nature Methods* (2008) 5:1005-1010]. For a chimera to produce a nominated fusion, the two cDNAs must be ligated at exon-exon boundaries. If the size of a typical exon is *L* bp, and *n* are the total number of reads failing PE alignment (roughly 14 million), the expected rate of chimera reads at exon-exon junctions produced would be (0.05×*n*)/*L*^2^. For example, if *L* = 100 bp, this predicts only 70 junction reads.

**Analysis of fusion detection pipeline and assignment of statistical significance**

We were able to use a statistical model to estimate false discovery rate (FDR) for nominating fusions with various junctional read-counts, detailed below. Our conclusion is that fusions supported by only one unique junctional read have a FDR that is too large to be considered as bona fide candidates. On the other hand, fusions supported by 4 junctional reads, such as the most abundant isoform of ESRRA-C11orf20, have a low estimated FDR, of the order 10^-6^.

*Details of FDR estimation:*

We compiled a table of read-pairs (read1,read2) where read1 maps to geneA and read2 maps to a constructed exon-exon junction between geneA and geneB; these were sub-grouped by junction offset in read2.

These were then filtered to ensure that the paired-reads mapped to complementary strands of the putative fusion transcript, since the two sequences produced by the paired-end sequencing procedure are derived from complementary strands of the DNA fragment: that is, that the strand orientation of the reads is either (+, –) or (–, +).

Although we did not do so for the analysis here, we note that a more stringent orientation filter could be applied:

• for pairs of the form (geneA, geneA-geneB), the read orientations should be (+, –)

• for pairs of the form (geneA, geneB-geneA), the read orientations should be (–, +).

Pairs of the form (geneA, geneA-geneB) with read orientation (–, +) are not supportive of real fusions, but can arise if there is homology between gene A and gene B, especially if in conjunction with a sequencing error (see Figure S2). Hence, such reads can be used to estimate a null model in which all fusion reads are explained by sequence homology. The count distribution for this null model and the alternative model (where read orientations are opposite but not further filtered) are:

| **gene-junction pair counts** | **1** | **2** | **3** | **4** | **5** |
| --- | --- | --- | --- | --- | --- |
| **alternative model** | 576 | 37 | 7 | 5 | 1 |
| **null model** | 93 | 2 | -- | -- | -- |

We compute the number of reads which failed to align as pairs:

• number of paired-reads where read1 failed to align = 4,420,340

• number of paired-reads where read2 failed to align = 3,399,371

• number of paired-reads where read1 and read2 map to different genes A and B = 677,528

• thus, number of reads that failed to align as pairs = 4,420,340 + 3,399,371 – (2 x 677,528) = 6,464,655

To develop the statistical estimates used for the FDR, consider geneA-geneB reads supported by only one unique read (from table above, there are 93 such in the null model, 576 in the alternative model). Under the null model, the probability of this event is estimated via the maximum likelihood estimator of a binomial(*n*=6,464,655, *p*). This gives the estimate *p* = 93/6,464,655. Under this estimate, consider

FDR = E(false_positives/all_positives)

for a pair called to be a real fusion when it is supported by one junctional read. This can be estimated by:

E(false_positives) / all_positives = 93/576 = 16%

Now, consider geneA-geneB reads supported by more than one unique read. Let *X* be the random variable representing the number of unique junctional reads exceeding one. Assume *X* has the Poisson distribution with rate parameter λ, and estimate λ under the null. Again, the simple maximum likelihood estimator estimates λ as *m*/*n*, where *m* is the number of times we observe *k*+1 reads, and *n* is total observations. Under this model, the only gene-junction counts that are non-zero are for *k* = 1 (table above), so *m* = 2, and we estimate λ as 2/*n*.

Using this estimate of λ, it is possible to compute a *p*-value for each observation of geneA-geneB fusion having *k*+1 unique junctional counts:

*p* = e^–λ^ λ*^k^* / *k*!

It is now possible to compute a *q*-value, or, the minimum FDR rate at which the genes with *k*+1 counts supporting them are true positives using the Benjamini-Hockberg step-wise FDR controlling procedure which rejects all hypotheses according to *p*-values in increasing order:

*p*_(1)_, *p*_(2)_, ..., *p*(*_m_*_–1)_, where *m* is the smallest number so *p*_(m)_ > *qm*/*n*.

We will approximate λ as 2/*n* (as discussed above); *n* = 6,464,655 (the non-aligning pairs), so *O*(10^6^); then λ = *O*(10^–6^).

Therefore, the single gene pair with count *k* = 4 will be rejected with

*q* = λ^4^/24 × 2/λ = λ^3^/12 = *O*(10^-18^).

The 4 gene pairs with *k* = 3 will be rejected with

*q* = λ^3^/6 × 5/λ = *O*(10^-12^).

The 7 gene pairs with *k* = 2 will be rejected with

*q* = λ^2^/3 × 12/λ = *O*(10^-6^).

The 36 genes with *k* = 1 will be rejected when

*q* = λ^1^ is less than 46*q*/*n* = 23*q*λ, that is for *q* = 1/23.

Note that these calculations assume that the measured counts are actually perfect, a questionable assumption. The precision in these *p* values is only good up to this assumption and so should only be taken as guidelines to significance.

**c) Fusion Candidate List**

The ranked list of fusion candidates is presented in Table S2.

The top ranked fusion candidate, PMS2L3-PRKRIP1, supported by 7 junctional reads (2 distinct junctions), appeared to be reporting on a true readthrough transcript at the time of our analysis and is now annotated as RefSeq LOC100630923. This fusion transcript is predicted to encode a protein corresponding to an N-terminally truncated variant of PRKRIP1. It is certainly possible that this readthrough could be a regulated event occurring at higher frequency in ovarian cancers.

We have not yet experimentally tested for any other fusion candidates besides ESRRA-C11orf20. ESRRA-C11orf20 is second on the list, with a total of 5 junctional reads (2 distinct junctions).

We note that one of the ESRRA-C11orf20 junctions, E2-C4, is supported by only one junctional read and is not considered statistically significant on its own. While in this case it was not a major issue, since the other junction was statistically significant, it points out that a more complex statistical model that could take into account multiple fusion isoforms between a pair of genes could have greater power for detecting gene fusions.

**d) ESRRA-C11orf20 Fusion Sequences**

Below we present the putative sequences for the three observed ESRRA-C11orf20 fusion transcript isoforms. Note that these were compiled based on the known sequences of the normal ESRRA and C11orf20 transcripts; while we have direct evidence for the junctional sequences, we do not know with certainty about sequences upstream of ESRRA exon 2 or downstream from our primers in C11orf20.

*Predicted sequences of ESRRA-C11ORF20 fusion transcripts*

The ESRRA portion is in black, the C11orf20 portion is in blue. The underlined sequences are directly supported by Sanger sequencing of products from RT-PCR.

>fusion_E2-C3

TCCTACAAGCAGCCGGCGGCGCCGCCGAGTGAGGGGACGCGGCGCGGTGGGGCGGCGCGG

CCCGAGGAGGCGGCGGAGGAGGGGCCGCCCGCGGCCCCCGGCTCACTCCGGCACTCCGGG

CCGCTCGGCCCCCATGCCTGCCCGACCGCGCTGCCGGAGCCCCAGGTGACCAGCGCCATG

TCCAGCCAGGTGGTGGGCATTGAGCCTCTCTACATCAAGGCAGAGCCGGCCAGCCCTGAC

AGTCCAAAGGGTTCCTCGGAGACAGAGACCGAGCCTCCTGTGGCCCTGGCCCCTGGTCCA

GCTCCCACTCGCTGCCTCCCAGGCCACAAGGAAGAGGAGGATGGGGAGGGGGCTGGGCCT

GGCGAGCAGGGCGGTGGGAAGCTGGTGCTCAGCTCCCTGCCCAAGCGCCTCTGCCTGGTC

TGTGGGGACGTGGCCTCCGGCTACCACTATGGTGTGGCATCCTGTGAGGCCTGCAAAGCC

TTCTTCAAGAGGACCATCCAGGAAAAGTCTTCCTCAATGTCATCACTCAATATTGCGAAG

CACATGCCCCATCGAGCCTACTGGGCAGAGCAGCAGAGCAGGCTGCCACTGCCCCTGATG

GAACTCATGGAGAATGAAGCTCTGGAAATCCTCACCAAAGCCCTCCGGAGCTACCAGTTA

GGGATCGGCAGGGACCACTTCCTGACTAAGGAGCTGCAGCGATACATCGAAGGGCTCAAG

AAGCGCCGGAGCAAGAGGCTGTACGTGAATTAAAAACGCCACCTTGGGCTCGAGCAGCGA

CCCGAACCAGCCCCGTGCCAGCCCGGTCCCCAGACCCAAGCCTGACCCCATCCGAGTGGA

ATTTGAGTCCTAAAGAAATAAAAGAGTCGATGCA

>fusion_E2-C4

TCCTACAAGCAGCCGGCGGCGCCGCCGAGTGAGGGGACGCGGCGCGGTGGGGCGGCGCGG

CCCGAGGAGGCGGCGGAGGAGGGGCCGCCCGCGGCCCCCGGCTCACTCCGGCACTCCGGG

CCGCTCGGCCCCCATGCCTGCCCGACCGCGCTGCCGGAGCCCCAGGTGACCAGCGCCATG

TCCAGCCAGGTGGTGGGCATTGAGCCTCTCTACATCAAGGCAGAGCCGGCCAGCCCTGAC

AGTCCAAAGGGTTCCTCGGAGACAGAGACCGAGCCTCCTGTGGCCCTGGCCCCTGGTCCA

GCTCCCACTCGCTGCCTCCCAGGCCACAAGGAAGAGGAGGATGGGGAGGGGGCTGGGCCT

GGCGAGCAGGGCGGTGGGAAGCTGGTGCTCAGCTCCCTGCCCAAGCGCCTCTGCCTGGTC

TGTGGGGACGTGGCCTCCGGCTACCACTATGGTGTGGCATCCTGTGAGGCCTGCAAAGCC

TTCTTCAAGAGGACCATCCAGGCTGCCACTGCCCCTGATGGAACTCATGGAGAATGAAGC

TCTGGAAATCCTCACCAAAGCCCTCCGGAGCTACCAGTTAGGGATCGGCAGGGACCACTT

CCTGACTAAGGAGCTGCAGCGATACATCGAAGGGCTCAAGAAGCGCCGGAGCAAGAGGCT

GTACGTGAATTAAAAACGCCACCTTGGGCTCGAGCAGCGACCCGAACCAGCCCCGTGCCA

GCCCGGTCCCCAGACCCAAGCCTGACCCCATCCGAGTGGAATTTGAGTCCTAAAGAAATA

AAAGAGTCGATGCA

>fusion_E2-C5

TCCTACAAGCAGCCGGCGGCGCCGCCGAGTGAGGGGACGCGGCGCGGTGGGGCGGCGCGG

CCCGAGGAGGCGGCGGAGGAGGGGCCGCCCGCGGCCCCCGGCTCACTCCGGCACTCCGGG

CCGCTCGGCCCCCATGCCTGCCCGACCGCGCTGCCGGAGCCCCAGGTGACCAGCGCCATG

TCCAGCCAGGTGGTGGGCATTGAGCCTCTCTACATCAAGGCAGAGCCGGCCAGCCCTGAC

AGTCCAAAGGGTTCCTCGGAGACAGAGACCGAGCCTCCTGTGGCCCTGGCCCCTGGTCCA

GCTCCCACTCGCTGCCTCCCAGGCCACAAGGAAGAGGAGGATGGGGAGGGGGCTGGGCCT

GGCGAGCAGGGCGGTGGGAAGCTGGTGCTCAGCTCCCTGCCCAAGCGCCTCTGCCTGGTC

TGTGGGGACGTGGCCTCCGGCTACCACTATGGTGTGGCATCCTGTGAGGCCTGCAAAGCC

TTCTTCAAGAGGACCATCCAGGGCTACCAGTTAGGGATCGGCAGGGACCACTTCCTGACT

AAGGAGCTGCAGCGATACATCGAAGGGCTCAAGAAGCGCCGGAGCAAGAGGCTGTACGTG

AATTAAAAACGCCACCTTGGGCTCGAGCAGCGACCCGAACCAGCCCCGTGCCAGCCCGGT

CCCCAGACCCAAGCCTGACCCCATCCGAGTGGAATTTGAGTCCTAAAGAAATAAAAGAGT

CGATGCA

**e) RT-PCR: Primers and Product Sizes**

Figure S3 shows the confirmation of the fusions by RT-PCR from the same pool RNA used for RNA-Seq, using two primer pairs. The products were cloned and sequenced, showing E2-C3 and E2-C4 fusions.

Figure 1C shows RT-PCR of positives from screens of individual patients using nested PCR primers (described in *Materials and Methods*); the inner primer pair is G1P2-FWD/F1-REV.

**Expected sizes of RT-PCR products for various fusion isoforms:**

| **primer pair** | **expected product (bp)** | | |
| --- | --- | --- | --- |
|  | **E2-C3** | **E2-C4** | **E2-C5** |
| G1P1-FWD / G2P1-REV | 515 | 435 | 368 |
| G1P2-FWD / G2P2-REV | 603 | 523 | 456 |
| G1P2-FWD / F1-REV | 507 | 427 | 360 |

*primers used in Figure S3:*

>G1P1-FWD

GGCATTGAGCCTCTCTACATCA

>G1P2-FWD

AAAGGGTTCCTCGGAGACAGAGA

>G2P1-REV

TCGATGTATCGCTGCAGCTCCTTA

>G2P2-REV

ACTCAAATTCCACTCGGATGGG

***2. Genomic Analysis***

**a) Overview of Sequencing Samples**

To assess genomic status of the C11orf20-ESRRA region, we originally made Illumina paired-end sequencing libraries using phi29-amplified DNA from four tissue samples: matched tumor and normal from two patients (tumors 2 and 3). We confirmed tumor-specific rearrangements by PCR in the phi29-amplified DNA but were unable to confirm these rearrangements in non-amplified material.

We prepared Illumina libraries from two fusion positive tumors (Tumors 1,2) and a matched PBMC normal sample from the patient of Tumor 2. The libraries were enriched for the target region by hybridization-selection with a bacterial artificial chromosome (BAC). The resulting enriched libraries were each sequenced on a separate lane of an Illumina GAII flowcell. Experimental details are in *Materials and Methods*.

**b) Sequencing Enrichment and Coverage of the Region Of Interest (ROI)**

We calculated the enrichment due to hybridization-selection, and the coverage (sequencing depth) for the region of interest (ROI).

*sizes in bp:*

total size = 2,897,310,462 (based on the number of non-N bases in the hg19 reference genome)

BAC ROI size = 166,356 (GenBank AP001453, coordinates 63995291 - 64161647 on hg19)

read-length = 80

Coverage = ROI reads × 2 × read-length / ROI size

*note:* BAC ROI read count includes counts of PE reads represented by distinct PE start and stop coordinates.

| **sample** | **internal ID** | **BAC ROI reads** | **coverage** | **average coverage** |
| --- | --- | --- | --- | --- |
| Patient 1 Tumor | 227742 | 1501128 | 744 | 883 |
| Patient 2 Tumor | 272383 | 1231214 | 1270 |
| Patient 2 PBMC (normal) | 321781 | 1644285 | 848 |

**c) Read Coverage Bias in Hybridization-Selected Libraries**

UHTS technologies are prone to uneven coverage, presumably due to biases resulting from library construction steps. In our case, we superimpose additional possibilities for bias due to the hybridization selection step and subsequent PCR amplification.

The sequencing depth shows over 100-fold variation at different positions in the ROI. As an example, we give below the profile of read coverage variation for the BAC-selected Patient 2 Normal sample. Note that the other tissue samples are known to contain segmental duplications and other differences from the reference genome, which introduce additional reasons for read coverage variation.

| **Variation in Number of Unique Reads by 10bp intervals (Normal PBMC)** | | | | | | |
| --- | --- | --- | --- | --- | --- | --- |
| **Align to Strand** | **Minimum** | **1st Quartile** | **Median** | **Mean** | **3rd Quartile** | **Maximum** |
| – | 0 | 176 | 468 | 500 | 767 | 2157 |
| + | 0 | 202 | 476 | 502 | 765 | 2359 |

(window: 6405300:6410420)

**d) Candidate rearranged regions from statistically anomalous read-pair distributions**

Briefly, read-pairs (read1, read2) with anomalous insert lengths were surveyed from each of the three samples and subjected to statistical analysis. We divided a 50 kb ROI (chr11:6405300-6410420) into a two-dimensional grid of 100 bp × 100 bp bins. A count was placed in bin (*x*, *y*) for each read-pair where: read1 aligned in bin *x*, the reverse-complement of read2 aligned in bin *y* which was a unique alignment in the ROI, and |*y* – *x*| > 1000. For each sample A, counts in a bin (*x*, *y*) were set to zero if sample B had any counts in the bin (*x*, *y*). This procedure served both as a discovery tool for anomalous read-pairs and a method for forming the null distribution of falsely identified candidate locations for anomalous read-pairs.

To discover anomalies in Tumor samples:

A = Patient 1 Tumor, B = Patient 2 PBMC (normal).

A = Patient 2 Tumor, B = Patient 2 PBMC (normal).

To assess the likelihood that a nominated count would arise from applying this approach to data with no genomic rearrangement, we performed this procedure on a null model, trying to discover anomalies separately in the following combination:

A = Patient 2 PBMC (normal), B = Patient 2 Tumor

The null distribution produced (*x*, *y*) count distributions with a maximum count of 4 in any bin. For tumor 2, the maximum bin count in the tumor did not exceed the maximum bin count in the null model. However, in tumor 1, the maximum bin count (observed in the areas where a rearrangement was subsequently confirmed) was 30. Similar results were obtained when we required the *x* alignment to be unique.

Under this null model, we consider the probability *p* of the null distribution of counts showing more than 4 counts in an (*x*, *y*) bin of the histogram. Using a simple binomial model with *n* = number of read-pairs in the experiment produces an estimate of *p* = o(1/*n*). Thus, if the observed frequency of bins with more than 4 counts >> o(1/*n*), the probability that observed empirical distribution was produced by the null model becomes vanishingly small.

If we conservatively say *n* (number of read-pairs, see table in section 2b above) is on the order of 10^5^, then under mild assumptions (i.e. a simple binomial model for counts), the *p*-value that the empirical counts would be observed is *p* = o(10^–5^). A Poisson model for the histogram counts would produce a still smaller *p*-value.

**e) Limited Assembly using Velvet**

We used the information from the above anomalous read-pair analysis to do a more restricted assembly using the *de novo* assembler, Velvet. For each sample, we ran through the following steps.

1) For each window of 300 bp, retrieve all orphan read-pairs in which one side mapped to the 300 bp window on the + strand.

2) Give these reads to Velvet as input, for assembly of contigs.

3) Filter to select contigs inconsistent with the reference genome

4) Select contigs generated from sample A and which have only reads from sample A mapping uniquely to them and which have a mate pair aligning uniquely to the genome.

5) Select for contigs with multiple mapping distinct read pairs.

6) Repeat the above for reads on the – strand.

For Tumor 1, this analysis found one contig in the ROI which coincided with our Sanger sequenced PCR product. No other contig with reads capable of explaining the ESRRA-C11orf20 fusion transcript were found (in either Tumor 1 or Tumor 2).

**f) Statistical Model for Copy Number Variation**

Pairs of reads which matched in an orientation consistent with the reference genome were used to estimate copy number. Multiple read pairs aligning to coordinates (*x*,*y*) could conceivably be PCR duplicates and were counted only once. For each bin, a sampling bias was estimated using the PBMC sample, and the ratio of tumor to normal reads was computed. An exact 95% confidence interval for this ratio was computed using the assumption of Poisson read distributions per bin, and the fact that if *X* and *Y* are independent Poisson random variables, *X*|*X+Y* has the binomial distribution.

***3. DNA rearrangements***

**a) Tumor 1 Breakpoint and Flanking Sequence from Three Sets of Primers**

**Sequences:**

In the FASTA sequences below, ESRRA sequence is in red, C11orf20 sequence is in black and/or underline. Note that 17 nucleotides at the junction are identical and could be assigned either to ESRRA or C11orf20.

>PCR1

ACTGAGGGTTCAAGGGGTTAGGTATGGGACTTGCCCAAGGTCATAAAGGTATGTGGTAGCCAGAGTCCCTGTTCGGCACA

GACCTGTTCTTTGCTGTCCTGGCCAGTGTTCCAGGCCTTGGGGACATAGCTGGGGCTGAAGCAGGGCTGTTTCTGCCCTC

AGGCAGTTTACATCCTGGCAGAGGGGAGAGCTGGGCAACAGTGAGTTGCACAGACTTGTCTTATTACCGCTGTGGTATGT

GCAGGAAGGGGAGGTGCTGGTTCTGAGGCTCCAGAGGGCTTGTCTTTTTTTTTTTTTTTTGAGACGGAGTCTCGCTTTGT

TGCCCAGGCTAGAGTCCgGTGGCGCGATCTCGGCTCAGTGCAAGCTCCGCCTCCCGGGTTCAAGCGATTCTCCTGCCTCA

GCCTCCCCAATAGCTGGGATTACAGGCACTTGCCAGCAAGCCCGGCTAATTTTTGTATTTTTAGTAGAGACGGGGGTTTC

ACCATGTTGGCCAGGTTGGTCTCGAACTCCTGACCTTGTGATCCGCCCACATTGGCCTCCCAAAGTGCTAGGATTACAGG

CATGAGCCACCGTGCCCGGCCAAGAGCTGTTCTTATTACCCCA

>PCR2

CTGGTTCTGAGGCTCCAGAGGGCTTGTCTTTTTTTTTTTTTTTTGAGACGGAGTCTCGCTTTGTTGCCCAGGCTAGAGTC

CGGTGGCGCGATCTCGGCTCAGTGCAAGCTCCGCCTCCCGGGTTCAAGCGATTCTCCTGCCTCAGCCTCCCCAATAGCTG

GGATTACAGGCACTTGCCAGCAAGCCCGGCTAATTTTTGTATTTTTAGTAGAGACGGGGGTTTCACCATGTTGGCCAGGT

TGGTCTCGAACTCCTGACCTTGTGATCCGCCCACATTGGCCTCCCAAAGTGCTAGGATTACAGGCATGAGCCACCGTGCC

CGGCCAAGAGCTGTTCTTATTACCCCATTTTACTGATGAAGAACTTGAGGGTCAGAGAGTGTCAGTCACTGGTCCTAAGT

GTCACACTGTGGGGCAGTCATTACTGGAACCATGTGGGTGGGGCTCTAAAGCCCAGGGTTGTAACCTCTGTCCCATGCTG

ACCCCAAGGCTGCACCAGGAGCTGGTCTCTGAAGGCCATTCCCTCTCACAAGGTGCCTACCCATCTCCTGTGCTCCAGGG

AAGGCCCAGGAGTCTGCGGATGACTAACCCTTTTTTCTCTTCTCCCAGAAAAGTCTTCCTCAATGTCATCACTCA

>PCR3

CTGATTGTGACATTCCAGCACGTTTTTTTTTTTTTTTTTGAGACAGTCTCGTGTGGTCGCCCAGGCTGGAGTGCAGTGGC

ACGATCTCGGCTCACTGCAAGCTCCGCCTCCCGGGTTCACACCATTCTCCTGCCTCAGCCTCCTGAGTAGCTGGGACTAC

AGGTGCCCGCCACCACGCCTGGCTAATTTTTTGTACTTTTAGTARAGACGGGGTTTCAGCGTGTTAGCCAAGATGGTCTT

GATTTCCTGACCTCGAGATCCACCTGCCTTGGACTCCCAAAGTGCTGGGATTACAGGCGTGAGCCACTGCTCCTGGCCAG

GTTTTTTTTTTTTTTTTTTTTTTTTTTTTGARATGGAGTTTTGCTCTTGTTGTCCAGGCTGGAGTGCAACGGCCTGCAGT

CGTGGTTCACTGCAACCTCTGCCTCCCGGGTTCAAGCCATTCACCTGCCTCARCCTCCCAAGTARCTGGGATTACAGGCG

CCTGCCACCATGCCCGGCTAATTTTTGTGTTTTTARTAGGGATGGGGTTTCACCATGTTGGCCAGGCTGGCCTCAAACTC

CTGACCTCAGGCGATCTGCCCTCCTCGGGCTTCCAAAGTGCTGGGATTATAGGTGTGAGCCACTGCACCCCGCCAATCCA

GCAAGTTTTAACTTGGCCAAAATCCACCAATCTTAAACTTTGTGCACCCTTCCCgCTCTGAAGAACAGTGAGCCAGCCGG

CCAGGGTGCGGGTATCTCCTACCTACCCTGGGGCCCCTCACTGTATGTTGACTATTGACAAATATTTATTGTGTGCTGGC

TGTGAATAGGACTTGTATATTGAGCACTTAGGTGTCATGAACCATGCTGGATGTTTTGACCATATTATCCCCTTTAATTC

TCACGACCCAACTCTGTGGGGCACTTTTACAGCTGGGAAACTGAGGGTTCAAGGGGTTAGGTATGGGACTTGCCCAAGGT

CATAAAGGTATGTGGTAGCCAGAGTCCCTGTTCGGCACAGACCTGTTCTTTGCTGTCCTGGCCAGTGTTCCAGGCCTTGG

GGACATAGCTGGGGCTGAAGCAGGGCTGTTTCTGCCCTCAGGCAGTTTACATCCTGGCAGAGGGGAGAGCTGGGCAACAG

TGAGTTGCACAGACTTGTCTTATTACCGCTGTGGTATGTGCAGGAAGGGGAGGTGCTGGTTCTGAGGCTCCAGAGGGCTT

GTCTTTTTTTTTTTTTTTGAGACGGAGTCTCGCTTTGTTGCCCAGGCTAGAGTCCgGTGGCGCGATCTCGGCTCAGTGCA

AGCTCCGCCTCCCGGGTTCAAGCGATTCTCCTGCCTCAGCCTCCCCAATAGCTGGGATTACAGGCACTTGCCAGCAAGCC

CGGCTAATTTTTGTATTTTTAGTAGAGACGGGGGTTTCACCATGTTGGCCAGGTTGGTCTCGAACTCCTGACCTTGTGAT

CCGCCCACATTGGCCTCCCAAAGTGCTAGGATTACAGGCATGAGCCACCGTGCCCGGCCAAGAGCTGTTCTTATTACCCC

ATTTTACTGATGAAGAACTTGAGGGTCAGAGAGTGTCAGTCACTGGTCCTAAGTGTCACACTGTGGGGCAGTCATTACTG

GAACCATGTGGGTGGGGCTCTAAAGCCCAGGGTTGTAACCTCTGTCCCATGCTGACCCCAAGGCTGCACCAGGAGCTGGT

CTCTGAAGGCCATTCCCTCTCACAAGGTGCCTACCCATCTCCTGTGCTCCAGGGAAGGCCCAGGAGTCTGCGGATGACTA

ACCCTTTTTTCTCTTCTCCCAGAAAAGTCTTCCTCAATGTCATCACTCA

**Primers:**

*PCR1:*

>T2F

TCACGACCCAACTCTGTGG

>T2R

CACTTAGGACCAGTGACTGACA

*PCR2:*

>ESRRA-FWD6

CTGGTTCTGAGGCTCCAGAG

> C11orf20-REV10

TGAGTGATGACATTGAGGAAGAC

*PCR3:*

>ESRRA-FWD10

CTGATTGTGACATTCCAGCACGT

>C11orf20-REV10

TGAGTGATGACATTGAGGAAGAC
